# Partially hydroxylated ultrathin iridium nanosheets as efficient electrocatalysts for water splitting

**DOI:** 10.1093/nsr/nwaa058

**Published:** 2020-04-08

**Authors:** Zifang Cheng, Bolong Huang, Yecan Pi, Leigang Li, Qi Shao, Xiaoqing Huang

**Affiliations:** College of Chemistry, Chemical Engineering and Materials Science, Soochow University, Suzhou 215123, China; Department of Applied Biology and Chemical Technology, Hong Kong Polytechnic University, Hong Kong, China; College of Chemistry, Chemical Engineering and Materials Science, Soochow University, Suzhou 215123, China; College of Chemistry, Chemical Engineering and Materials Science, Soochow University, Suzhou 215123, China; College of Chemistry, Chemical Engineering and Materials Science, Soochow University, Suzhou 215123, China; College of Chemistry, Chemical Engineering and Materials Science, Soochow University, Suzhou 215123, China

**Keywords:** iridium, nanosheet, 2D material, hydroxylation, overall water splitting

## Abstract

Ultrathin two-dimensional (2D) materials have attracted considerable attention for their unique physicochemical properties and promising applications; however, preparation of freestanding ultrathin 2D noble metal remains a significant challenge. Here, for the first time, we report use of a wet-chemical method to synthesize partially hydroxylated ultrathin Ir nanosheets (Ir-NSs) of only five to six atomic layers’ thickness. Detailed analysis indicates that the growth confinement effect of carbon monoxide and the partially hydroxylated surface play a critical role in formation of the ultrathin structure. The ultrathin Ir-NSs exhibit excellent performance for both the hydrogen evolution reaction and oxygen evolution reaction in a wide pH range, outperforming the state-of-the-art Pt/C and IrO_2_, respectively. Density-functional theory calculations reveal that the partial hydroxylation not only enhances the surface electron transfer between Ir-sites and intermediate O-species, but also guarantees efficient initial activation of bond cleavage of H-O-H for first-step H_2_O splitting. This, ultimately, breaks through barriers to full water splitting, with efficient electron transfer essentially maintained.

## Teaser

This work reports the first-time synthesis of partially hydroxylated ultrathin iridium nanosheets with only 5-6 atomic layer thickness, which exhibit excellent catalytic performance for overall water splitting.

## INTRODUCTION

Electrocatalytic water splitting is a key technology for energy conversion and storage, which can transfer the electricity from intermittent renewable energy (e.g. solar energy and wind energy) into storable chemical energy (H_2_) [1–4]. However, the sluggish kinetics of electrode reactions, including the hydrogen evolution reaction (HER) and oxygen evolution reaction (OER) can decrease the energy conversion efficiency and hinder the application of related devices [5,6]. To date, Pt-based and Ir-based compounds are the state-of-the-art HER and OER catalysts, respectively, with excellent intrinsic performance [7–9]. However, their high cost and scarcity greatly hinder large-scale applications. Reducing usage of such metals yet improving their performance is critical for development of water splitting technology [10,11].

Since the successful exfoliation of graphene in 2004, ultrathin 2D materials have attracted tremendous research attention [12]. They often possess unusual properties compared to their bulk counterparts and thus have potential for a wide range of applications [13–17]. In terms of 2D metal-based electrocatalysts, ultrathin structures usually have an extremely high ratio of catalytic active surfaces, improving atom utilization and reducing metal usage [18,19]. Coordinatively unsaturated metal sites at the surface, as well as the unique edge structure, also may contribute to higher intrinsic activity [20,21]. However, unlike intrinsic layered materials (e.g. layered double hydroxides), it is thermodynamically unfavorable for most noble metals to form 2D morphology because of the high surface energy, making synthesis extremely difficult [22]. Using capping agents and templates, several well-defined 2D noble metal nanosheets have been successfully synthesized [23–26]; however, synthesis of freestanding Ir-based ultrathin nanosheet has scarcely been reported. Therefore, developing a facile method to synthesize ultrathin Ir-based materials is desirable to optimize their properties and extend their applications.

Herein, we report a facile wet-chemical method to synthesize freestanding ultrathin Ir nanosheets (simplified as Ir-NSs) of only five to six atomic layers’ thickness. Mechanism study shows that the introduced formic acid plays a critical role in formation of the ultrathin sheet-like morphology, which decomposes to carbon monoxide (CO) and hydrogen (H_2_) during the reaction process. On one hand, CO and H_2_ can facilitate reduction of Ir precursor, but, on the other hand, CO strongly bound to the metal basal (111) facets acts as a surface-confining agent to promote the preferred 2D growth. Further characterization indicates that the partially hydroxylated surface of Ir-NSs is also beneficial to formation and preservation of layered ultrathin NS. Benefiting from a large specific surface area and largely exposed coordinatively unsaturated surface sites of ultrathin NSs, the obtained Ir-NSs exhibit superior electrocatalytic activity for both OER and HER in a wide pH range. The OER mass activity of Ir-NSs is 2.8 and 4.7 times higher than that of IrO_2_ in 0.5 M H_2_SO_4_ and 1 M KOH, respectively. The excellent HER performance also outperforms Pt/C, with a 50-mV-improvement in overpotential (at 10 mA/cm^2^) in alkaline conditions. For overall water splitting, only 1.586 V and 1.575 V are required to achieve 10 mA/cm^2^ in 0.5 M H_2_SO_4_ and 1 M KOH, respectively, much lower than that required for commercial Pt||IrO_2_. Density-functional theory (DFT) calculations reveal strong p-d coupling between surface Ir-5d bands and p-orbitals of electronegative −OH groups, increasing the electronic occupation of Ir-5d bands and lowering the Ir-5d band center to prevent Ir-O over binding. Meanwhile, the surface electronic orbital distribution further promotes p-p endwise overlapping. Such electronic activity trends guarantee substantial competitively preferred H-O-H scissoring-dominated alkaline HER and OER catalysis.

## RESULTS AND DISCUSSION

**Figure 1.**
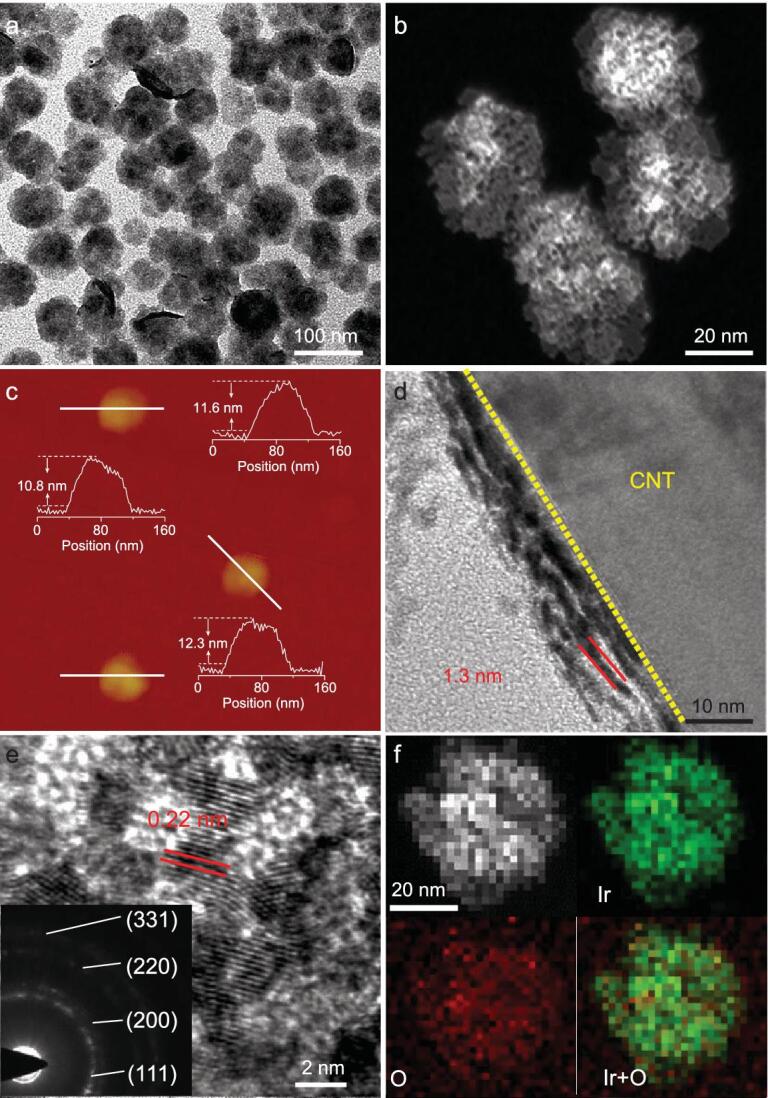
(a) TEM, (b) HAADF-STEM, and (c) AFM images of Ir-NSs. (d) The TEM image of CNT supported Ir-NSs perpendicular to the TEM grid. (e) HRTEM image and (f) HAADF-STEM-EDS elemental mappings of Ir-NSs. The inset in (e) shows the SAED pattern of Ir-NSs.

The Ir-NSs were synthesized by a wet-chemical method (see details in Methods). A typical transmission electron microscopy (TEM) image (Fig. 1a) shows that the products with a nearly round-sheet morphology are well dispersed and have an average size of ∼57 nm (Supplementary Fig. 1). We can clearly see from the high-angle annular dark-field scanning TEM (HAADF-STEM) (Fig. 1b) that Ir-NSs are assembled by multiple individual sheets, with an average total thickness of ∼11 nm determined by atomic force microscopy (AFM) (Fig. 1c). To further characterize the multi-layer structure, Ir-NSs were supported on carbon nanotubes (Fig. 1d and Supplementary Fig. 2). The average thickness of a single sheet was found to be ∼1.3 nm, about five to six atomic layers thick, proving the ultrathin nature of Ir-NSs. High-resolution TEM (HRTEM) analysis was also conducted (Fig. 1e). It is noted that the sheet-like structure can be gradually destroyed when exposed to the electron beam radiation (Supplementary Fig. 3), which also supports the ultrathin nature. The lattice spacing of the destroyed Ir-NSs is 0.22 nm, consistent with the spacing of the (111) plane of face-centered cubic (*fcc*) Ir. The concentric circles in SAED pattern correspond to the (111), (200), (220) and (311) facets of *fcc* Ir, respectively. Therefore, we could conclude that *fcc* Ir is the major phase in the destroyed Ir-NSs. Furthermore, STEM energy dispersive X-ray spectroscopy (EDS) elemental mapping (Fig. 1f) shows uniform distribution of O element within the Ir-NSs, indicating the existence of oxidized Ir species.

**Figure 2.**
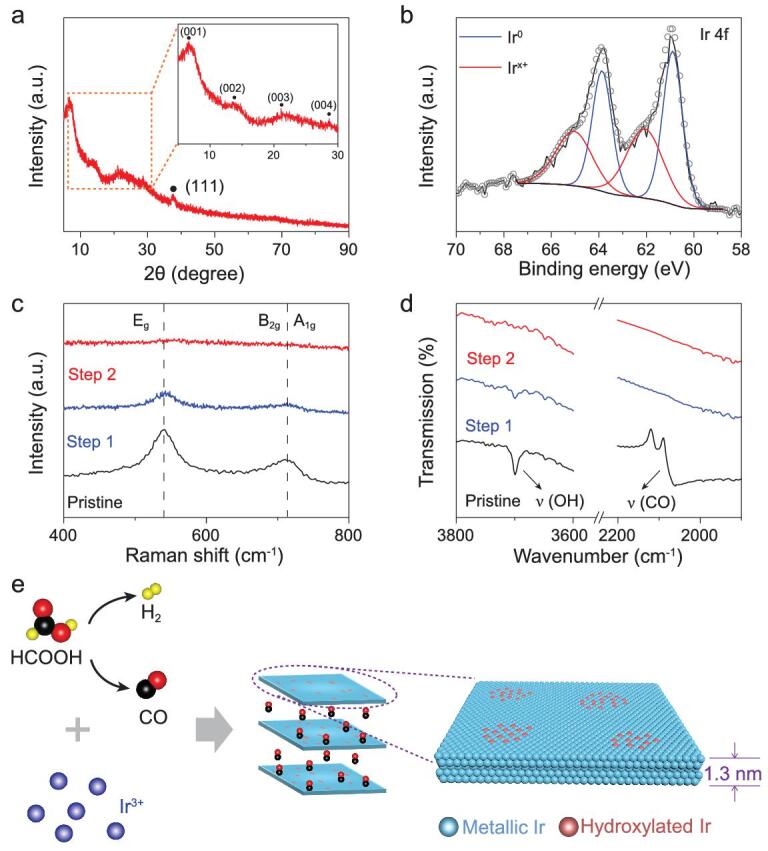
(a) PXRD pattern and (b) Ir 4f XPS spectrum of Ir-NSs. (c) Raman spectra and (d) FTIR spectra of Ir-NSs at different heating steps under Ar. Step 1, 2: temperature reaching 160 and 600°C, respectively. (e) A schematic showing the synthesis mechanism and structural model of Ir-NSs.

To further explore the structure characteristics of Ir-NSs, additional methods were used. Powder X-ray diffraction (PXRD) patterns of Ir-NSs (Fig. 2a) show only one peak located after 30°, which can be assigned to the (111) plane of *fcc* Ir. Considering the 2D ultrathin nature of Ir-NSs, we infer that Ir-NSs have (111) facet as the basal plane, which is parallel to the substrate. To support our deduction, Ir-NSs were loaded on carbon black to expose other facets (Supplementary Fig. 4). The diffraction peaks of Ir-NSs shift to lower angles compared with the typical *fcc* Ir (JCPDS card No. 06–0598), indicating lattice expansion in Ir-NSs. Interestingly, there is also a series of diffraction peaks before 30° with 2θ = 6.9°, 13.8°, 20.8° and 27.8°, corresponding to the (001) orientated layered structure with a basal spacing of 1.28 nm [27], which can be ascribed to the diffraction of an individual NS [28]. X-ray photoelectron spectroscopy (XPS) (Fig. 2b) shows that apart from the typical metallic Ir (Ir^0^) with binding energy (BE) of 60.9 and 63.9 eV, there is another Ir species with BE of 62.0 and 65.0 eV, which is 0.7 eV higher than that of the reported IrO_2_ (referred as Ir^x+^) [29,30]. Combining the results with H_2_ desorption area on a cyclic voltammetry (CV) curve, it is reasonable to infer that the surface of Ir NSs is partially oxidized (Supplementary Fig. 5). To understand the nature of the species Ir^x+^, Raman spectroscopy and Fourier-Transform infrared (FTIR) spectroscopy measurements were conducted. As shown in Fig. 2c, Ir-NSs exhibit peaks at 541 cm^−1^ and 712 cm^−1^, which arise from the typical Ir-O vibration [30]. Moreover, FTIR spectra (Supplementary Fig. 6) exhibit an obvious peak at ∼3700 cm^−1^, corresponding to the metal connected hydroxyl group (M-OH) [31]. Therefore, we deduce that the Ir^x+^ at Ir-NSs could be Ir oxyhydroxide species (IrOOH) [32]. Considering the intrinsic layered structure of IrOOH, the hydroxylated surface can reduce the surface energy and contribute to formation and preservation of layered Ir-NSs.

In addition, from the FTIR spectrum, surface-adsorbed CO was also detected at ∼2060 cm^−1^ [33]. Gas chromatography measurement (Supplementary Fig. 7) of the post-synthesis atmosphere also confirms the presence of CO and H_2_ during the reaction process, which could come from decomposition of formic acid (HCOOH → CO + H_2_O; HCOOH →CO_2_ + H_2_). Therefore, it is reasonable to infer that the released H_2_ and CO from formic acid may contribute to reduction of Ir precursor (Supplementary Fig. 8), and CO is responsible for directing formation of ultrathin NS by binding to the (111) planes of metallic Ir [23,34,35].

Thermogravimetric analysis (TGA) was carried out to gain deeper understanding of the unique structure. As displayed in Supplementary Fig. 9a, a total of ∼30% weight loss is observed when the temperature increases from room temperature to 600°C under an Ar atmosphere. In detail, two main weight losses occur at 160°C and 430°C, called step 1 and step 2. Products at these two steps were collected for further analysis. PXRD patterns (Supplementary Fig. 9b) show that the layered structure with expanded lattice breaks down at step 1. Then the typical *fcc* Ir phase is reformed on reaching step 2. According to the Raman spectra (Fig. 2c), the signal of Ir-O vibration decreases (step 1) and finally disappears (step 2). This result matches well with the FTIR spectra (Fig. 2d): the absorption band of Ir-OH also decays (step 1) and finally disappears (step 2) during the heating process. The consistency in the trend further proves that Ir^x+^ and O coexist in the form of Ir-OH, which gradually breaks down when the temperature keeps increasing. According to the FTIR spectra, the adsorbed CO is completely released at step 1. Therefore, the weight loss at step 1 mainly results from release of adsorbed CO, residual solvent and reduction of Ir-OH. Then the left Ir-OH further decomposes at step 2, leading to the second weight loss. From all the results above, the formation mechanism and structural characteristics of ultrathin Ir-NSs can be summarized (Fig. 2e). During the solvothermal reaction, formic acid decomposes to produce H_2_ and CO, which are typical reducing agents and contribute to reduction of the Ir precursor. Because of the confinement effect of CO, Ir species grow over the (111) plane in two dimensions. Meanwhile, the surface is partially hydroxylated, forming a stable ultrathin 2D structure of only five to six atomic layers’ thickness. In addition, the hydroxylated surface may lead to tensile strain, which is largely enhanced by the ultrathin nature [36]. As a result, obvious lattice expansion is observed (Supplementary Fig. 10).

**Figure 3.**
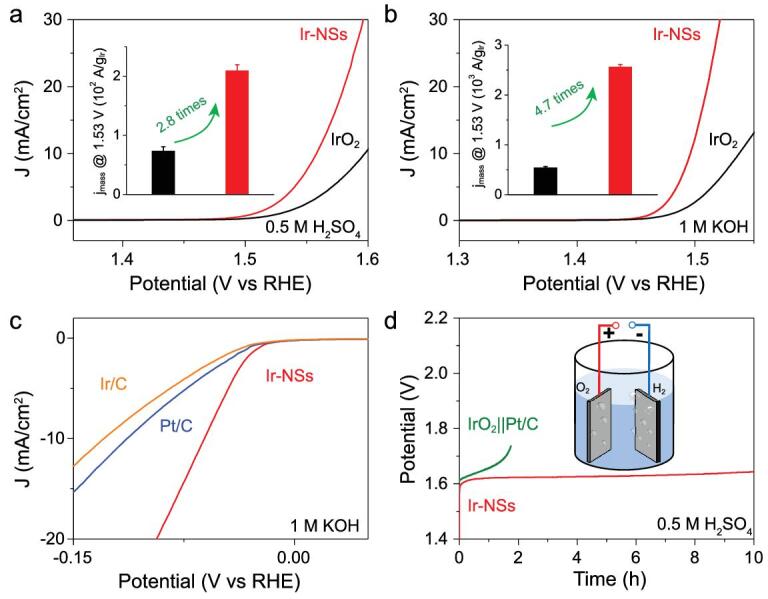
Polarization curves for OER in (a) 0.5_ _M H_2_SO_4_ and (b) 1 M KOH. The insets show the corresponding mass activity at 1.53 V_RHE_. (c) Polarization curves for HER in 1 M KOH. (d) CP curves for overall water splitting in 0.5 M H_2_SO_4_ at a constant current density of 5 mA/cm^2^. The inset shows the schematic diagram for electrocatalytic overall water splitting.

Electrochemical tests were carried out to evaluate the OER performance. To remove the side effect of poly(vinylpyrrolidone), surfactant-free Ir-NSs were used and the loading amount of Ir was fixed at 15 μg/cm^2^ for each test (Supplementary Figs 11–13). Ir-NSs deliver superior activity with an overpotential of 328 and 266 mV at 10 mA/cm^2^ in 0.5 M H_2_SO_4_ and 1 M KOH, respectively, which are both ∼40 mV lower than that of commercial IrO_2_ (Fig. 3a and b). In terms of mass activity, this is 209 and 2561 A/g_Ir_ in 0.5 M H_2_SO_4_ and 1 M KOH, which is 2.8 and 4.7 times higher than that of commercial IrO_2_, respectively. The OER tafel slope of Ir-NSs is 45.4 mV/dec in 0.5 M H_2_SO_4_ and 29.1 mV/dec in 1 M KOH (Supplementary Fig. 14), much smaller than that of commercial IrO_2_ (50.8 and 41.4 mV/dec), indicating highly improved OER kinetics for Ir-NSs. In addition, the enhanced activity of Ir-NSs could also be proved by the much lower interfacial charge-transfer resistance, indicating fast electron transport during the electrocatalytic process (Supplementary Fig. 15). Besides the high activity, the durability of Ir-NSs was explored with a chronoamperometry (CP) test in 0.5 M H_2_SO_4_, from which it can maintain almost constant potential for ∼10 h at 5 mA/cm^2^, whereas the activity of IrO_2_ completely decayed after only 4 h (Supplementary Fig. 16).

The HER activity of Ir-NSs was also evaluated. It was found that Ir-NSs also exhibit superior HER performance. In detail, Ir-NSs show similar activity to that of Pt/C in 0.5 M H_2_SO_4_ (Supplementary Fig. 17). More importantly, Ir-NSs outperform commercial Pt/C and Ir/C in 1 M KOH, with a 50-mV-improvement in overpotential (at 10 mA/cm^2^) (Fig. 3c). Therefore, Ir-NSs may act as both anode catalyst and cathode catalyst for overall water splitting. As displayed in Supplementary Fig. 18a and b, only 1.586 V and 1.575 V potentials are needed to reach 10 mA/cm^2^ in 0.5 M H_2_SO_4_ and 1 M KOH, respectively, much lower than for commercial IrO_2_||Pt/C (1.612 V and 1.633 V). Besides the reduced potential, Ir-NSs also possess ∼10 h stability at 5 mA/cm^2^ in 0.5 M H_2_SO_4_, much better than that of IrO_2_||Pt/C (<2 h, Fig. 3d).

**Figure 4.**
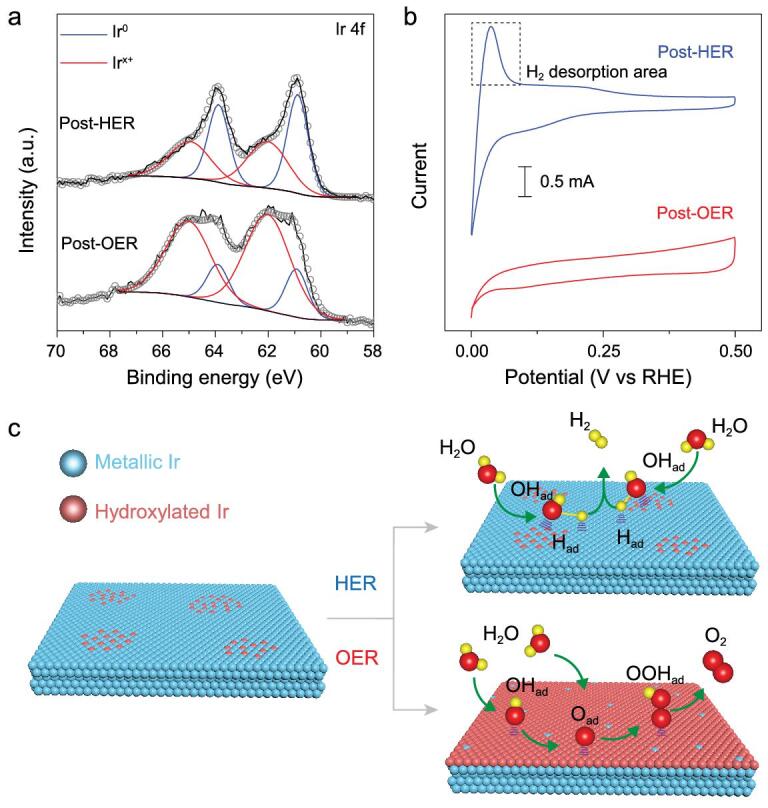
(a) XPS spectra and (b) CV curves of Ir-NSs after HER and OER. (c) Structural models of Ir-NSs for OER and HER catalysis.

After OER and HER, Ir-NSs were collected for characterization, with PXRD patterns indicating maintenance of the layered structure and metallic *fcc* phase (Supplementary Fig. 19). Additionally, XPS analysis (Fig. 4a) proves preservation of the surface chemical states (Ir^0^/Ir^x+^ = 52/48) after HER. The obvious H_2_ desorption area on the CV curve (Fig. 4b) also indicates maintenance of metallic Ir at the surface. Therefore, it is reasonable to deduce that the partially hydroxylated surface can be largely maintained during HER (Fig. 4c). Considering the unique advantages of metal-metal hydroxide systems for alkaline HER, the excellent HER activity of the Ir-NSs could be attributed to their partially hydroxylated surfaces [9,37]. On the contrary, the surfaces of Ir-NSs are largely oxidized during OER as indicated by the XPS spectrum and CV curve (Fig. 4a and b). Combining the PXRD result, we may infer formation of surface amorphous IrO_x_H_y_ during OER (Fig. 4c), which can act as superior active species [10,38].

**Figure 5.**
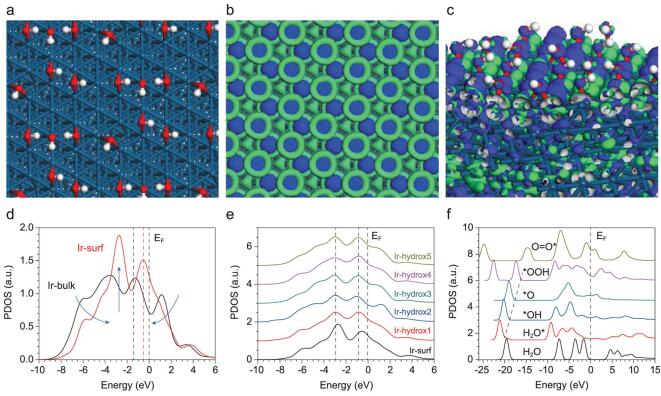
(a) Structural configurations of Ir-surface with partial hydroxylation. (b, c) The real spatial 3D orbital contour plots of pristine Ir-surface and partially hydroxylated Ir-surface, respectively. (d) PDOSs of Ir-5d band between surface and bulk states. (e) The PDOSs line-up of Ir-5d bands within different hydroxylated sites and pristine Ir-surface. (f) PDOSs of O-2p bands of O-intermediates during OER process.

We interpreted the high performance of the HER and OER given by the system. The relaxed surface shows that the hydroxyl groups are mostly two-fold coordinated, rather than three-fold pyramid or single dangling configurations. This implies that the Ir partial hydroxylation is meta-stable and optimal for O-species desorption (Fig. 5a). The active bonding and anti-bonding orbitals near the Fermi level (E_F_) are illustrated. The pristine Ir-surface has evidently shown the on-site isolated electron-localization center, indicating site-to-site electron transfer barriers (Fig. 5b). The surface electronic orbital distribution of partial hydroxylation shows that the site-to-site d-d electron-transfer forbidden feature has been suppressed. With surface hydroxylation, the surface Ir-sites are electron-rich centers, which enlarge the electron transfer rate between surface Ir-Ir-sites and Ir-O (Fig. 5c). The projected density of states (PDOSs) analysis of surface Ir-5d bands is illustrated. We confirm that the surface Ir-5d band denotes substantially higher electronic activity relative to the bulk states (Fig. 5d). With surface partial hydroxylation, the dominant twin-peak feature of surface Ir-5d band has been downshifted; in particular the first dominant peak has shifted from E_V_-0.5 eV to E_V_-1.0 eV (E_V_ = 0 for E_F_). The modified surface Ir-5d band centers are pinned and turn to be broadened. Such a feature optimizes the overlapping with O-2p orbitals of intermediates for better prevention of over-oxidation, resulting in deactivation of surface Ir-sites (Fig. 5e). From the PDOSs for O-2p band evolution of H_2_O adsorption and O-intermediate species, the linear scaling trend of O-2p sigma-band promotion has been preserved, which is guaranteed by the efficient electron transfer between surface Ir-5d and adsorbing intermediates (Fig. 5f).

**Figure 6.**
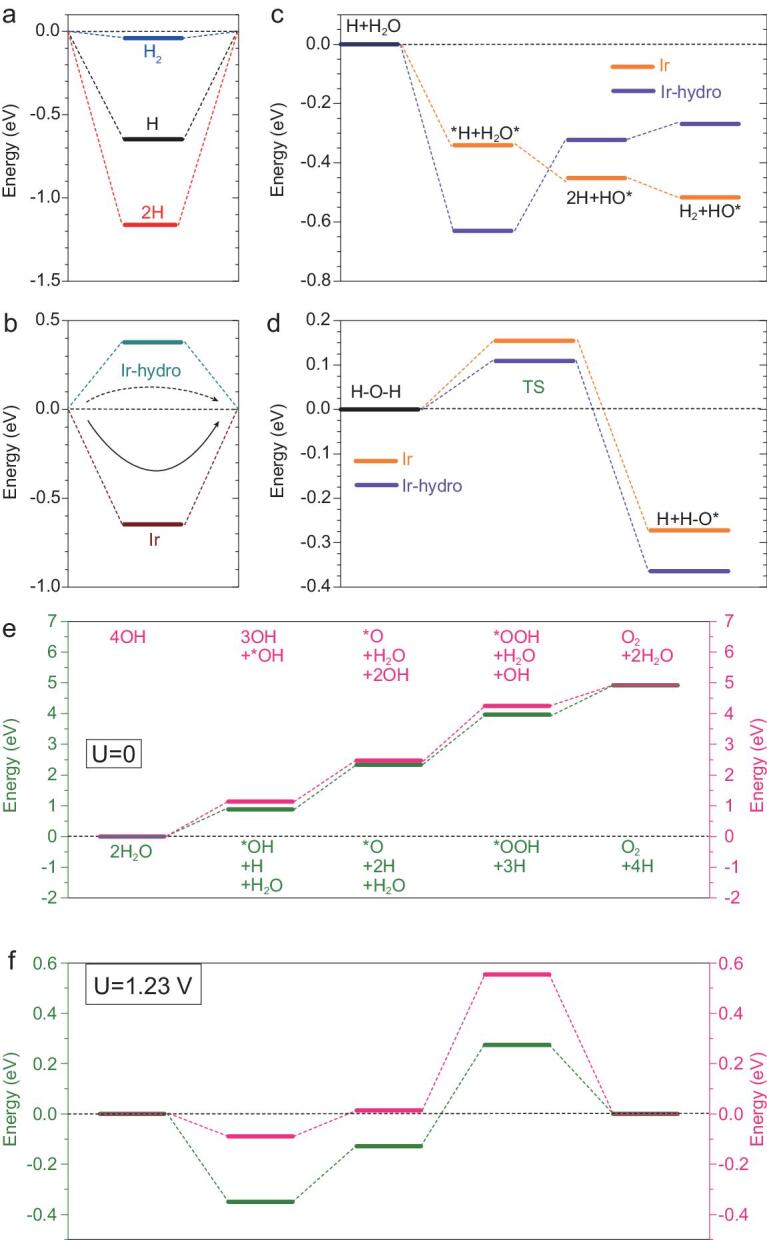
(a) H-adsorption energy of H, 2H, and H_2_ on the pristine Ir-surface. (b) H-adsorption energy comparison between pristine Ir- and hydroxylated surface. (c) Alkaline HER pathways for pristine Ir and hydroxylated surface. (d) H_2_O-splitting transition state barrier comparison. (e) The pathways of both four-electron based acidic and alkaline OER at U = 0 V. (f) The OER pathways are summarized at U = 1.23 V.

We move on to the energetic properties and pathways of both HER and OER on the pristine Ir and partially hydroxylated surfaces. For the HER on the pristine Ir-surface, H-adsorption is energetically favorable with −0.65 eV gained and the 2H-adsorption denotes a −1.18 eV level. Formation of H_2_ is also efficient at almost close to the thermoneutral line, with minor adsorption energy of −0.04 eV (Fig. 6a). The H-adsorption between pristine and fully hydroxylated Ir-surfaces was compared, with the contrast denoting that HER preferentially occurs at pristine Ir-surface. This indicates that the partially hydroxylation exposed Ir-surface preserves efficient HER (Fig. 6b). For the alkaline pathway, the contrast illustrates that energetically favored H_2_O adsorption on the Ir-hydroxylated surface results in an evident barrier to transformation into 2[(H^+^)+e^−^]+OH (Fig. 6c). Transition-state comparison of H_2_O splitting shows different capabilities of H-O-H bond cleavage, which potentially directs the H_2_O splitting on the Ir-hydroxylated surface (Fig. 6d). The trend in H_2_O splitting confirms an energetically favorable OER within both acidic and alkaline conditions. For the U = 0 V potential, both OER pathways demonstrate a similar energy level line-up (Fig. 6e). For U = 1.23 V, both OER pathways show the same potential determining step occurring at formation of ^*^OOH with theoretically estimated overpotential of 0.27 eV and 0.51 eV for the acidic and alkaline conditions, respectively (Fig. 6f). Therefore, both electronic and energetic properties have been discussed and consistently confirm the prominent overall H_2_O-splitting performance given by the partially hydroxylated Ir-surface system.

## CONCLUSION

In summary, we have successfully prepared ultrathin Ir-NSs with partially hydroxylated surfaces by a facile wet-chemical method. CO released from formic acid acts not only as a reducing agent but also as a surface confining agent, contributing to the sheet-like morphology. The hydroxylated surface reduces the surface energy, promoting formation and preservation of the ultrathin layered structure. The unique partially hydroxylated surface is maintained after HER and is mainly oxidized to amorphous IrO_x_H_y_ species after OER, which leads to enhanced HER and OER activities, respectively. DFT calculations reveal that electronegative OH modification induces a downshifting trend of Ir-5d band with band center pinned, preserving the Ir electroactivity. The competitively preferred H-O-H scissoring-dominated alkaline HER and OER originate from a substantially robust efficient electron transfer rate. With partial hydroxylation, the suppressed and broadened surface Ir-5d band guarantees electron transfer for both OER and HER without substantial electron-trapping from over-oxidation or (Ir-O)-over-binding-induced deactivation. We believe that partially hydroxylated ultrathin Ir-NSs can act not only as a high-efficiency water splitting catalyst but also as a model for exploring the properties of 2D metallic materials.

## METHODS

### Preparation of Ir-NSs and surfactant-free Ir-NSs

For the typical preparation of Ir-NSs, IrCl_3_ · H_2_O (12 mg) and PVP (20 mg) were dispersed in NMP/formic acid (6 mL/2 mL) mixed solution by sonication in an ultrasonic bath. The resulting homogeneous mixture was transferred into a 20 mL teflon-lined stainless steel and heated at 100°C for 5 h. The products were collected via centrifugation and washed with ethanol (1 mL) and acetone (8 mL) three times. The synthesis of surfactant-free Ir-NSs was the same as that of Ir-NSs, except that the PVP was replaced by carbon black (15 mg) to allow Ir-NSs to grow directly on carbon black.

### Electrochemical measurements

A three-electrode cell was used to conduct the electrochemical measurements by CHI 660E, in which a saturated calomel electrode (SCE) was used as the reference electrode, a carbon rod electrode was used as the counter electrode and a glassy carbon electrode (GCE) (diameter: 5 mm; area: 0.196 cm^2^) was used as the working electrode. Surfactant-free Ir-NSs (3.6 mg, 14.01 wt%, measured by TGA test) were dispersed in 1 mL solvent containing 20 μL of water, 970 μL of isopropanol and 10 μL of 5 wt% Nafion to form a homogeneous ink by sonication for 30 min (0.5 mg_Ir_/mL). The catalyst ink (6 μL, 3 μg of Ir) was then loaded onto a GCE. The reference electrode SCE was calibrated using a reversible hydrogen electrode (RHE). Two Pt electrodes were cleaned and used as working electrode and counter electrode. Calibration was conducted in the H_2_ saturated electrolyte by linear-sweep voltammetry (LSV). The resulting potential is the potential of zero net current. In this work, the potential of zero net current was found at −0.270 V vs. SCE electrode in 0.5 M H_2_HSO_4_ and −1.053 V vs. SCE in 1 M KOH (Supplementary Fig. 20).

$$\begin{equation*} \begin{array}{@{}*{1}{l}@{}} {{\rm{E}}\,{\rm{vs}}.\,{\rm{RHE}} {\,=\,} {\rm{E}}\,{\rm{vs}}.\,{\rm{SCE}} {\,+\,} 0.270\,{\rm{V}}\left( {0.5\,{\rm{M}}\,{{\rm{H}}_2}{\rm{S}}{{\rm{O}}_4}} \right)}\\ {{\rm{E}}\,{\rm{vs}}.\,{\rm{RHE}} {\,=\,} {\rm{E}}\,{\rm{vs}}.\,{\rm{SCE}} {\,+\,} 1.053\,{\rm{V}}\left( {1\,{\rm{M}}\,{\rm{KOH}}} \right)} \end{array}\end{equation*}$$

The OER measurements were carried out in N_2_-saturated 0.5 M H_2_SO_4_ and 1 M KOH. The CV tests were carried out between +1.0 and +1.7 V at 50 mV/s for 20 cycles to activate catalysts. LSV tests were recorded in the same potential range with a scan rate of 5 mV/s with 95% iR compensation.

The HER measurements were carried out in N_2_-saturated 0.5 M H_2_SO_4_ and 1 M KOH. The CV tests were carried out between +0.1 and −0.15 V at 50 mV/s for 20 cycles to activate catalysts. LSV tests were recorded in the same potential range with a scan rate of 5 mV/s with 95% iR compensation.

The overall water splitting measurements were carried out in N_2_-saturated 0.5 M H_2_SO_4_ and 1 M KOH. The CV tests were carried out between +1.0 and +1.7 V at 50 mV/s for 20 cycles to activate catalysts. LSV tests were recorded in the same potential range with a scan rate of 5 mV/s with 95% iR compensation.

The CV test for observing H_2_ desorption was carried out in H_2_-saturated 0.5 M H_2_SO_4_. The CV tests were recorded between 0 and 0.5 V at a scan rate of 100 mV/s.

The CP tests for OER and overall water splitting were measured in 0.5 M H_2_SO_4_ at a constant current density of 5 mA/cm^2^.

The Nyquist plots for OER were recorded at 1.55 V vs. RHE in N_2_-saturated 0.5 M H_2_SO_4_ and 1 M KOH.

### DFT calculations

We chose the simplified rotationally invariant DFT+U calculations [39] within CASTEP code [40] to calculate the electronic and energetic properties. The algorithm of Broyden-Fletcher-Goldfarb-Shannon was selected for ground state geometry optimization. The cutoff energy of plane-wave basis sets for total energy calculations was set to 750 eV. The PBE exchange-correlation functional was selected for DFT+U calculations. To improve the convergence quality of transition metal compound system, the ensemble DFT by Marzari *et al.* [41] was used during the electronic-minimization process.

The substrate Ir-surface model was built based on the bulk *fcc*-Ir crystal, in which the surface system is built six Ir layers thick, with a range of 150 atoms sized 5 × 5 × 1. Almost 40% portions of hydroxyl groups were manually attached on the Ir-surface to simulate the partial hydroxylation. Considering the cost of DFT computation, Monkhost-Pack reciprocal space integration was performed using Gamma-center-off special k-points with mesh of 2 × 2 × 2 [42], guided by the initial convergence test. With these settings, the overall total energy for each step converges to <5.0 × 10^−7 ^eV per atom. The Hellmann-Feynman forces on the atom were converged to <0.001 eV/Å.

Ir, O and H norm-conserving pseudopotentials were generated using the OPIUM code in the Kleinman-Bylander projector form [43], and non-linear partial core correction [44] and a scalar relativistic averaging scheme [45] were used to treat the mixed valence Ir spin-orbital coupling effect. We chose the projector-based (5*d*, 6*s*, 6*p*), (2*s*, 2*p*) and (1*s*) states to reflect the valence states of Ir, O and H atoms, respectively. The RRKJ method was chosen for optimization of the pseudopotentials [46].

## Supplementary Material

nwaa058_updated_supplemental_file
