# Supplementary material for: Partially hydroxylated ultrathin iridium nanosheets as efficient electrocatalysts for water splitting
Source: Natl Sci Rev. 2020 Apr 8;7(8):1340–8. doi: 10.1093/nsr/nwaa058 (PMC8288892; doi:10.1093/nsr/nwaa058)
Supplement: nwaa058_updated_supplemental_file [file nwaa058_updated_supplemental_file.pdf]

Supplementary Information for

Partially Hydroxylated Ultrathin Iridium Nanosheets as Efficient Electrocatalysts for Water Splitting

Zifang Cheng,[1] Bolong Huang,[2] Yecan Pi,[1] Leigang Li, [1] Qi Shao[1] and Xiaoqing Huang[*1]

[1]College of Chemistry, Chemical Engineering and Materials Science, Soochow University, Jiangsu 215123, China. *E-mail: hxq006@suda.edu.cn

[2]Department of Applied Biology and Chemical Technology, Hong Kong Polytechnic University, Hung Hom, Kowloon, Hong Kong SAR, China.

Materials and Methods

Chemicals: Iridium (III) chloride hydrate ($IrCl_3 \cdot H_2O$, 99.9%,) was purchased from Alfa Aesar. Poly(vinylpyrrolidone) (PVP, average M.W. 58000, K29-32), N-methyl-2-pyrrolidone (NMP 99%) and formic acid (HCOOH, 98%) were purchased from J&K Scientific Ltd. Carbon nanotube (CNT, multiwall, D*L 110-170 nm*5-9 μm) was purchased form Sigma-Aldrich. Carbon black (X72R) was purchased from Vulan. All the chemicals were used as received without further purification. The water (18 MΩ·cm) used in all experiments was prepared by passing through an ultra-pure purification system (Aqua Solutions).

Characterization: The TEM samples were prepared by dropping ethanol dispersion of Ir-NSs onto carbon-coated copper grids using pipettes and dried under ambient condition. Low magnification transmission electron microscopy (TEM) was conducted on a HITACHI HT7700 transmission electron microscope at an accelerating voltage of 120 kV. High-magnification TEM (HRTEM) and scanning TEM (STEM) were conducted on a FEI Tecnai F20 transmission electron microscope at an accelerating voltage of 200 kV. Powder X-ray Diffraction (PXRD) pattern was collected on X' Pert-Pro MPD diffractometer (Netherlands PANalytical) with a Cu Kα X-ray source (λ = 1.540598 Å). Thermogravimetric analysis was performed on SII TG/DTA 6300 thermogravimetric analyzer. The loading amount of catalysts was analyzed by the inductively coupled plasma optical emission spectrometer (ICP-OES, Varian 710-ES). Fourier transform infrared spectra were recorded on a ProStar LC240 spectrometer. Raman spectra were recorded on a Horiba HR800 Raman spectrometer using the 633 nm laser as the excitation source. X-ray photoelectron spectra (XPS) were collected with an SSI S-Probe XPS Spectrometer..

Supplementary Figures

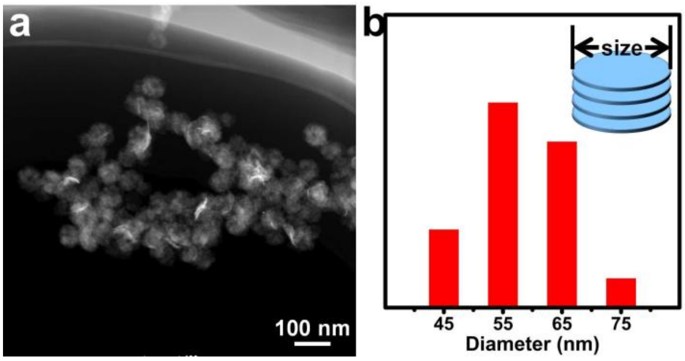

Supplementary Figure 1. (**a**) HAADF-STEM image, and (**b**) size distribution of Ir-NSs.

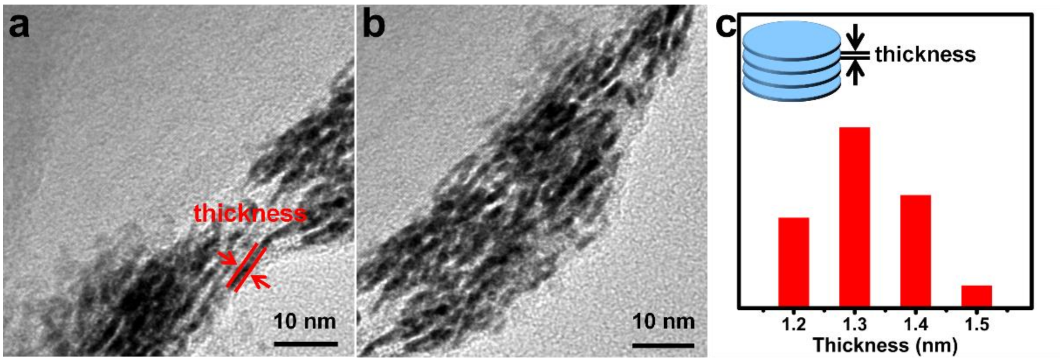

Supplementary Figure 2. (**a**, **b**) TEM images of carbon nanotube supported Ir-NSs and (**c**) thickness distribution of individual sheet in Ir-NSs.

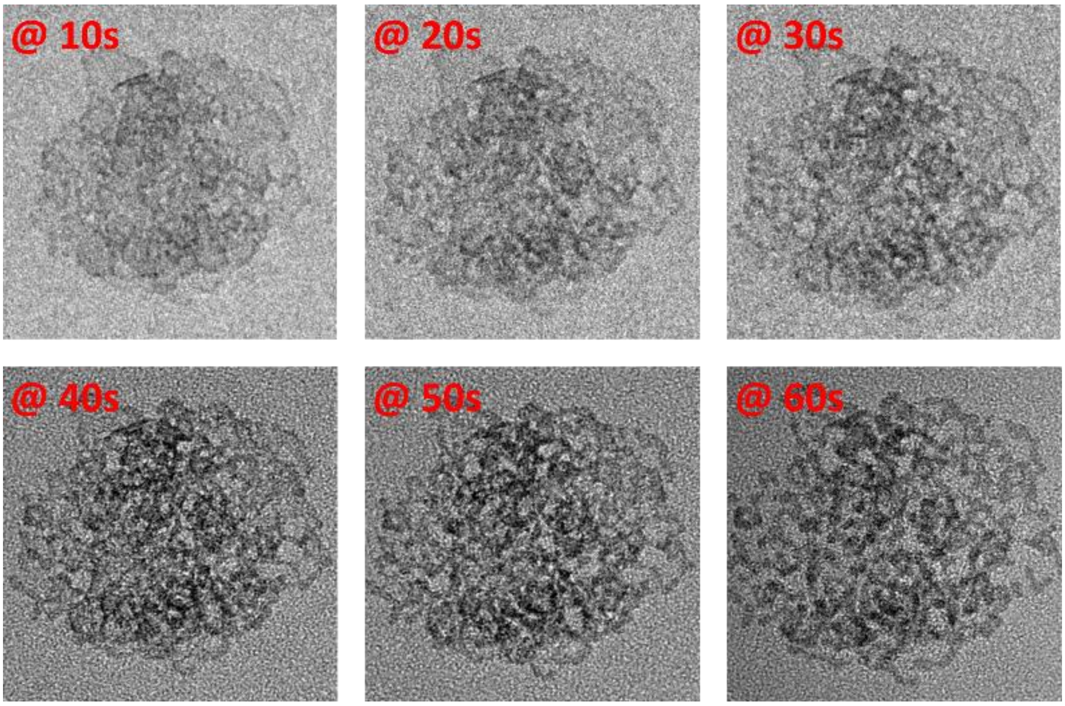

Supplementary Figure 3. TEM images of Ir-NSs irradiated under e-beam for different time.

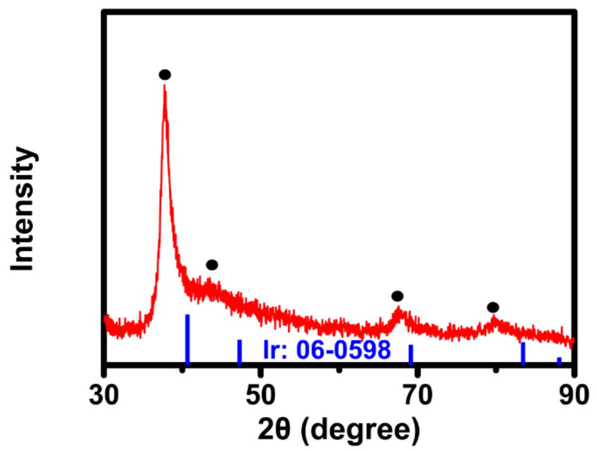

Supplementary Figure 4. PXRD parttern of Ir-NSs loaded on carbon black.

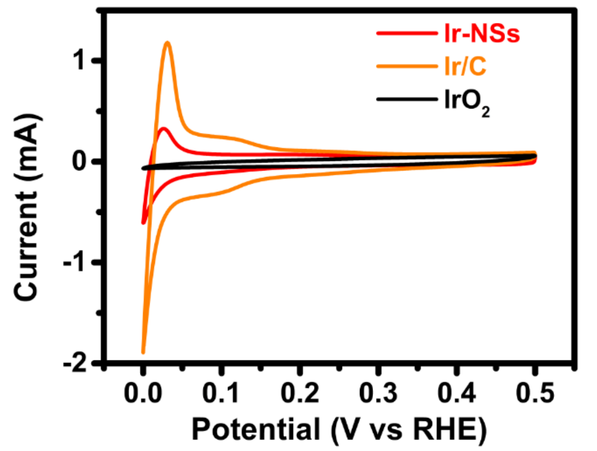

Supplementary Figure 5. CV curves collected at 100 mV/s in the potential range of 0 to 0.5 V vs RHE in 0.5 M H_2SO_4.

Note for Supplementary Figure 5: Ir/C possesses an obvious area for H_2 desorption while IrO_2 does not. Ir-NSs also have the H_2 desorption area, indicating the presence of metallic species on surface.

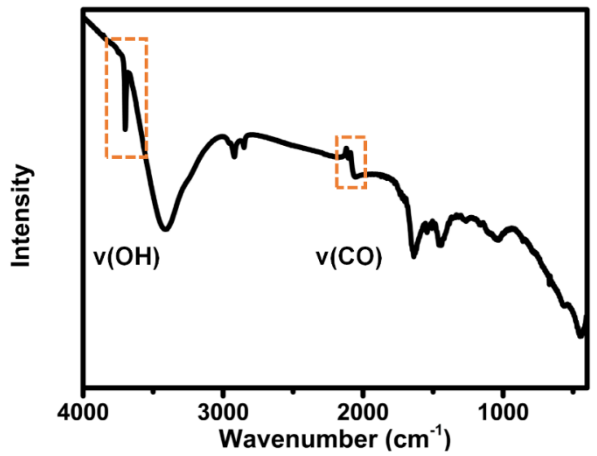

Supplementary Figure 6. FTIR spectra of Ir-NSs. The adsorbtion band at ~3700 cm^{-1} can be assigned to the Ir connected hydroxide group. The adsorbtion band at ~2060 cm^{-1} can be assigned to the absorbed CO on Ir surface.

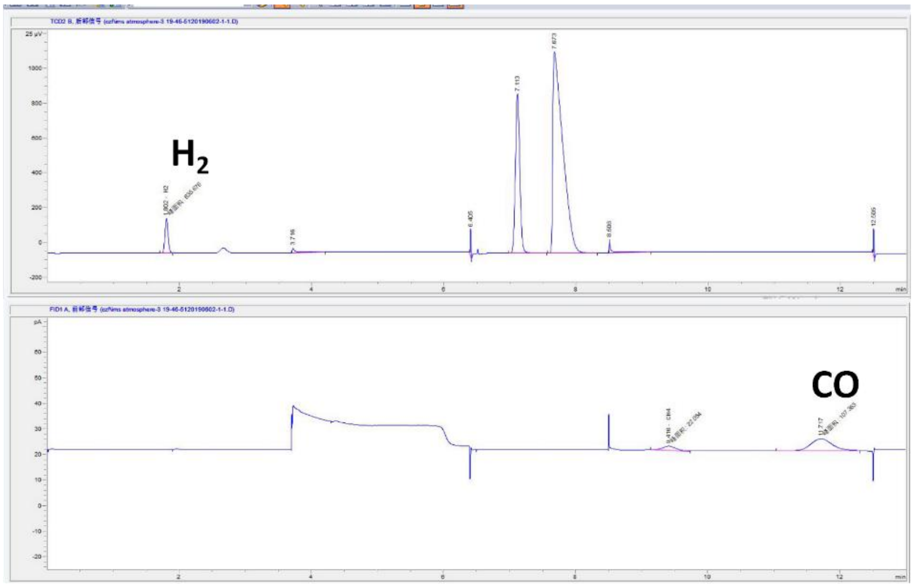

Supplementary Figure 7. Gas chromatograph showing the released H_2 and CO during reaction process.

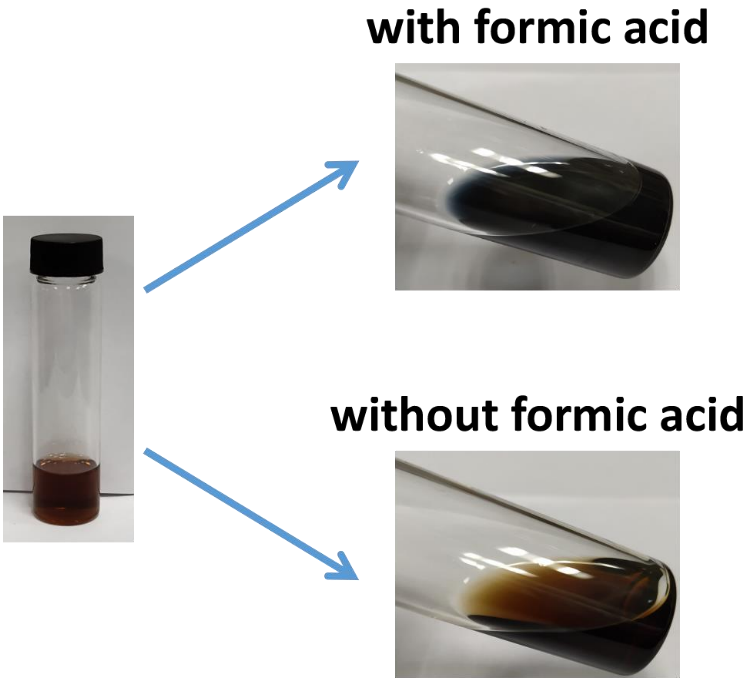

Supplementary Figure 8. Photographs of reaction mixture before heating and products obtained with and without addition of formic acid. Ir-NSs are dark blue when formic acid is added. No solid product is obtained without formic acid and the yellowish-brown color indicates Ir precursor was not reduced.

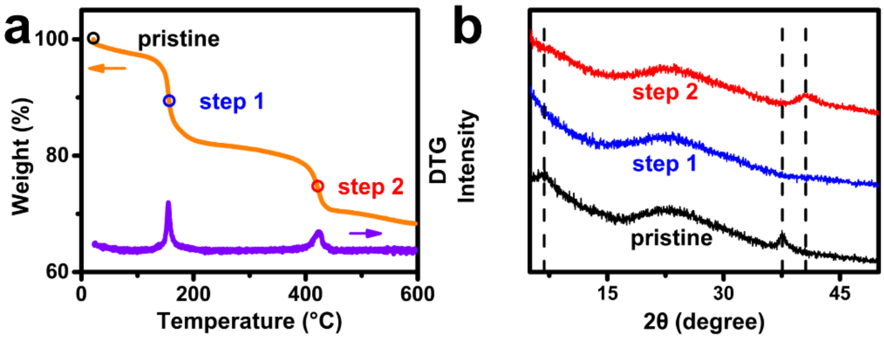

Supplementary Figure 9. (a) TGA and DTG curves of Ir-NSs. Data were collected under flowing Ar using a temperature ramp of 5 °C/min. Step 1, 2: temperature reaches 160 °C and 430 °C, respectively. (b) PXRD spectra of Ir-NSs at different steps of the heating process.

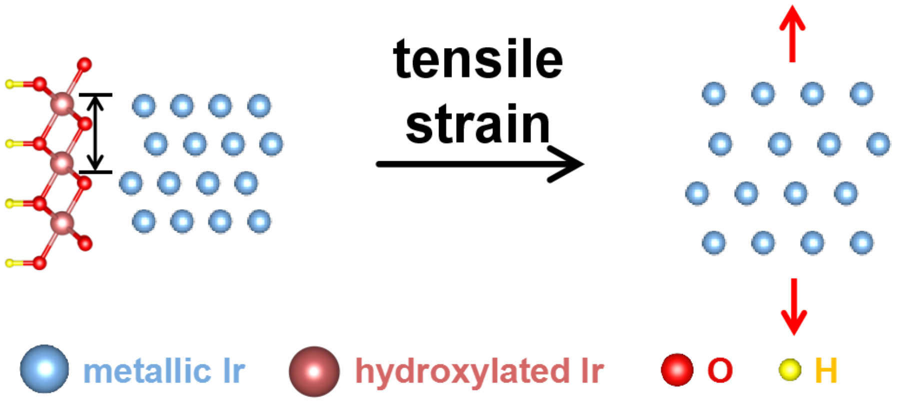

Supplementary Figure 10. Schematic illustrating lattice expansion in Ir-NSs.

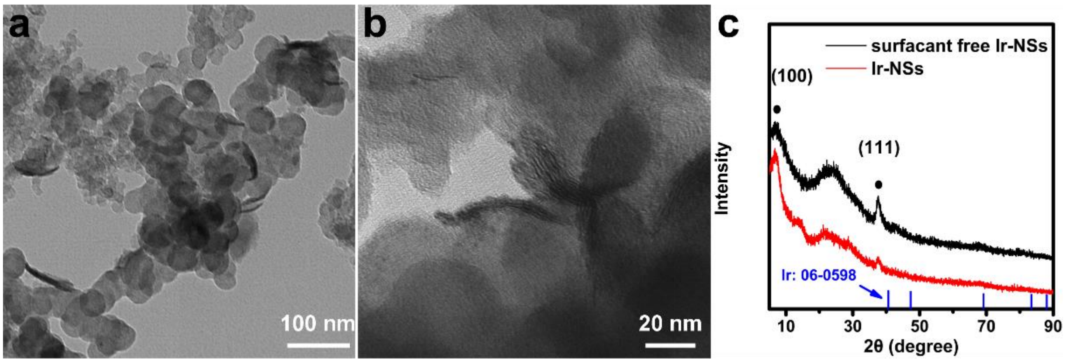

Supplementary Figure 11. (a, b) TEM images of surfactant free Ir-NSs and (c) PXRD pattern of Ir-NSs and surfactant free Ir-NSs.

Note for Supplementary Figure 11: Surfactant free Ir-NSs can keep the structure of multilayer ultrathin nanosheets. PXRD parttern also exhibits the same (100) and (111) peaks as Ir-NSs, indicating the maintenance of layered structure and expanded lattice. Therefore surfactant free Ir-NSs possess the same structure characteristic of Ir-NSs.

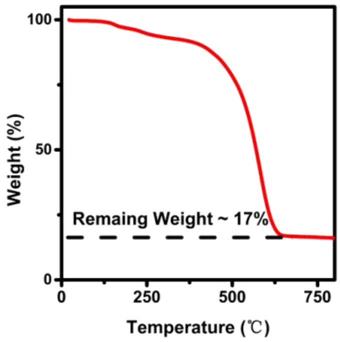

Method	Mass fraction of Ir (%)
TG	14.03
ICP-OES（dissolved in aqua regia under ambient conditions）	7.21
ICP-OES（dissolved in aqua regia under 100 ℃）	9.41

Supplementary Figure 12. TGA curves for Ir fraction determination and calculated Ir mass fraction (%) in surfactant free Ir-NSs by different methods. Data were collected under flowing air using a temperature ramp of 10 °C/min.

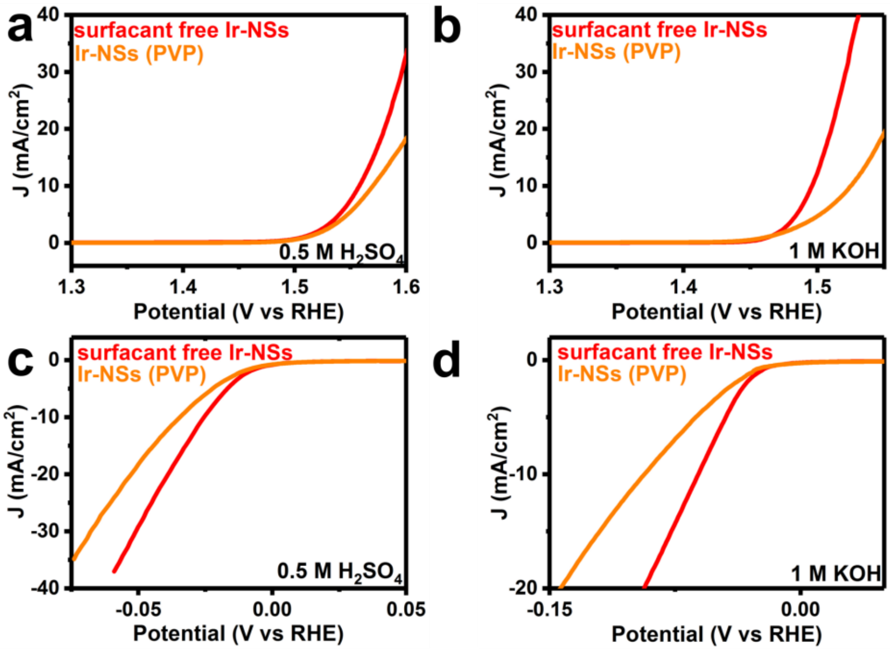

Supplementary Figure 13. Polarization curves of Ir-NSs (PVP) for OER in (a) 0.5 M H2SO4 and (b) 1 M KOH; for HER in (c) 0.5 M H2SO4 and (d) 1 M KOH.

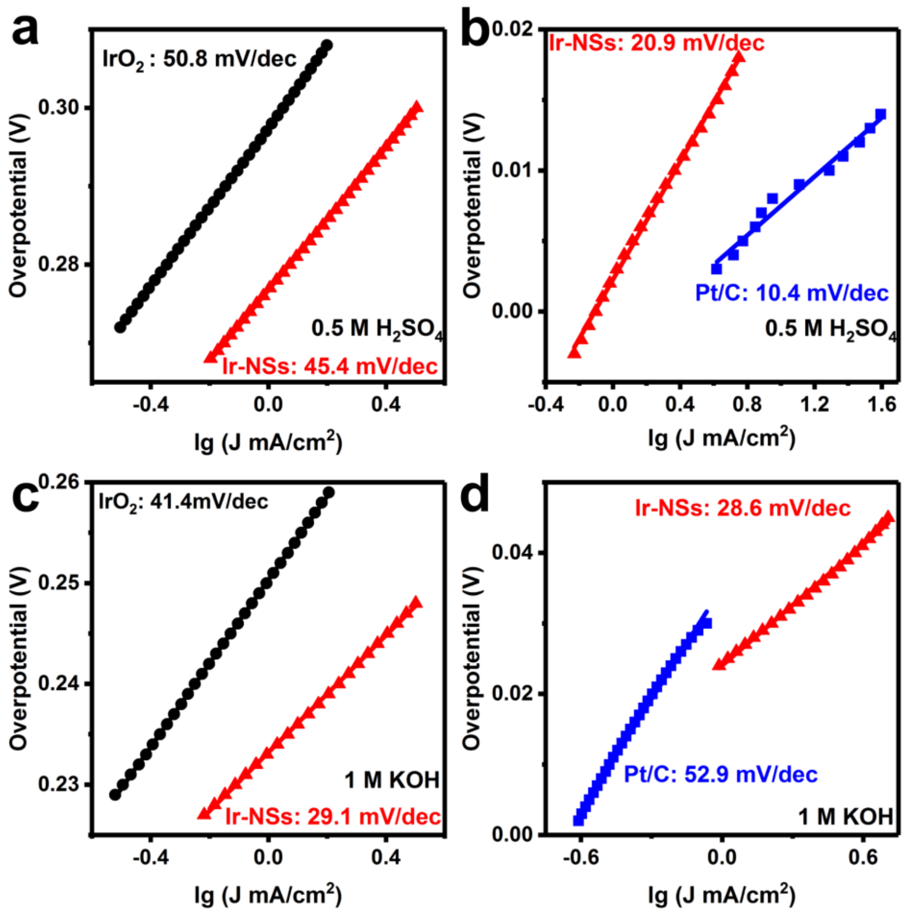

Supplementary Figure 14. OER tafel slope curves of Ir-NSs and IrO_2 in (a) 0.5 M H_2SO_4 and (c) 1 M KOH. HER tafel slope curves of Ir-NSs and Pt/C in (b) 0.5 M H_2SO_4 and (d) 1 M KOH.

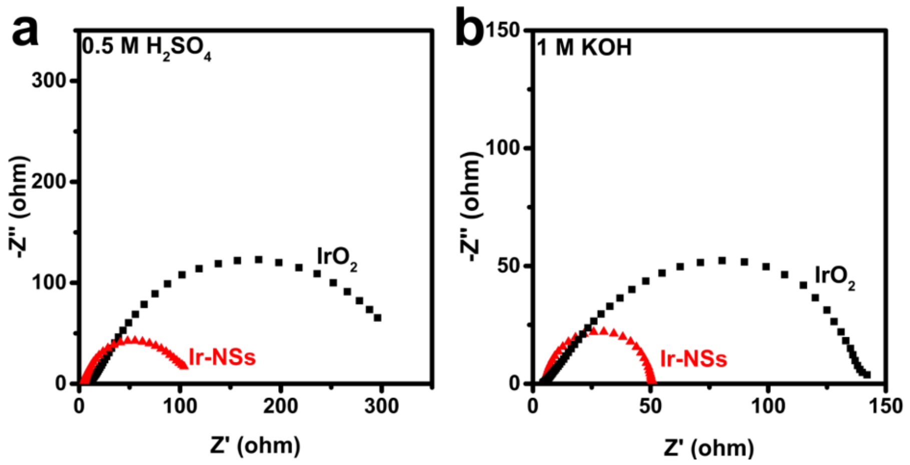

Supplementary Figure 15. Nyquist plots (recorded at 1.55V vs RHE) of Ir-NSs and IrO_2 for OER in (a) 0.5 M H_2SO_4 and (b) 1 M KOH.

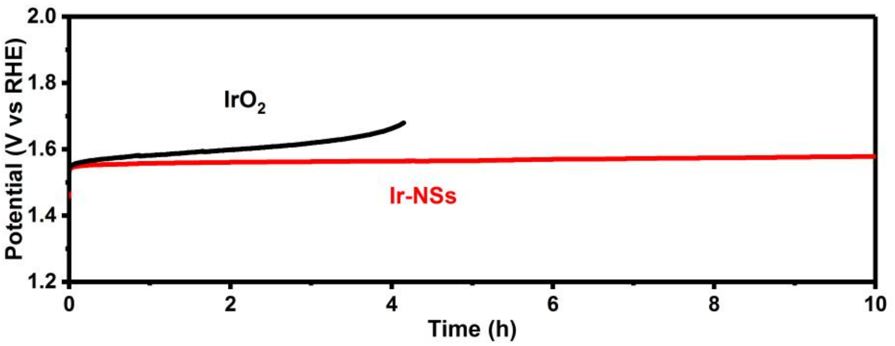

Supplementary Figure 16. OER CP curves of Ir-NSs and IrO_2 in 0.5 M H_2SO_4 at a constant current density of 5 mA/cm^2.

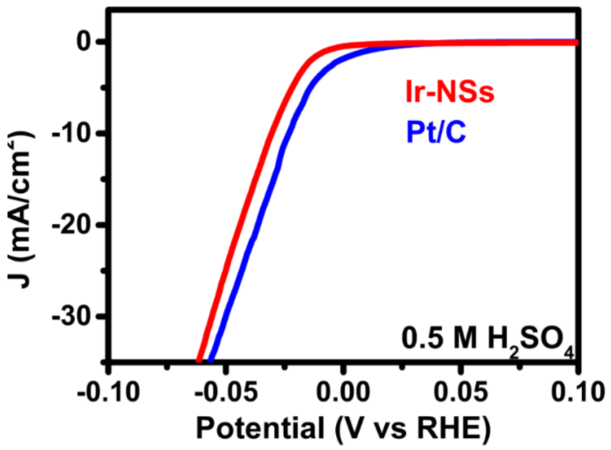

Supplementary Figure 17. HER polarization curves of Ir-NSs and Pt/C in 0.5 M H_2SO_4.

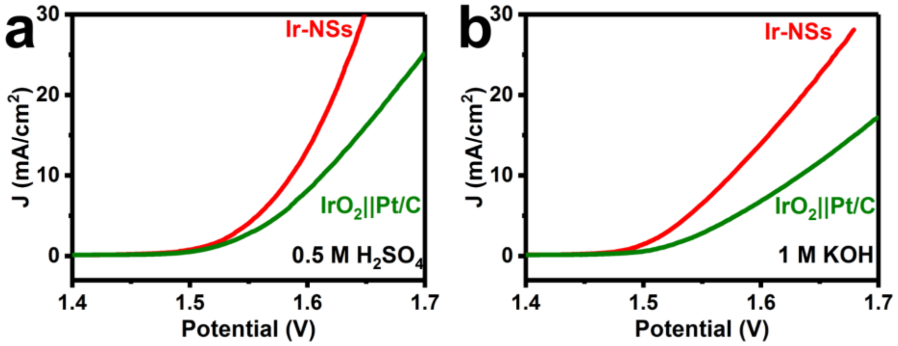

Supplementary Figure 18. Polarization curves for overall water splitting of Ir-NSs and IrO_2 ‖ Pt/C in (a) 0.5 M H_2SO_4 and (b) 1 M KOH.

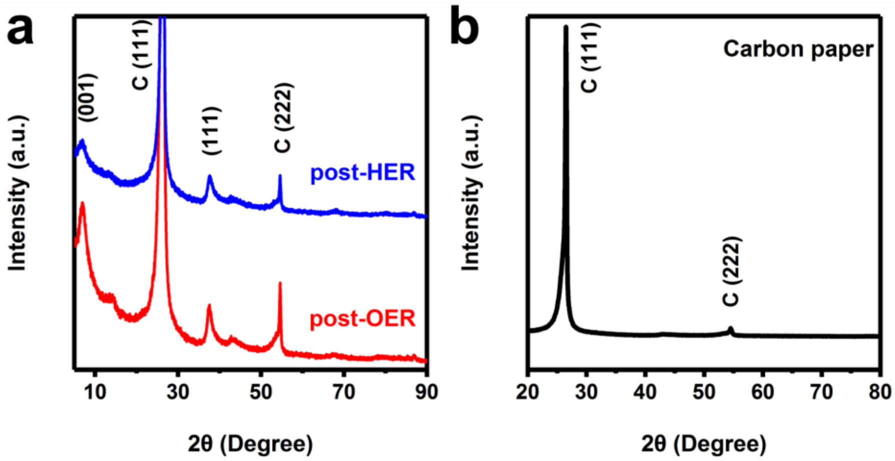

Supplementary Figure 19. PXRD curves of Ir-NSs after HER and OER (a) and pure carbon paper (b).

Note for Supplementary Figure 19: In order to collect the XRD data of the post reaction sample, carbon paper was used as the electrode. Catalyst ink was dropped on the carbon paper and then went through the electrochemical test. After that, carbon paper along with the loaded catalyst was characterized by XRD together.

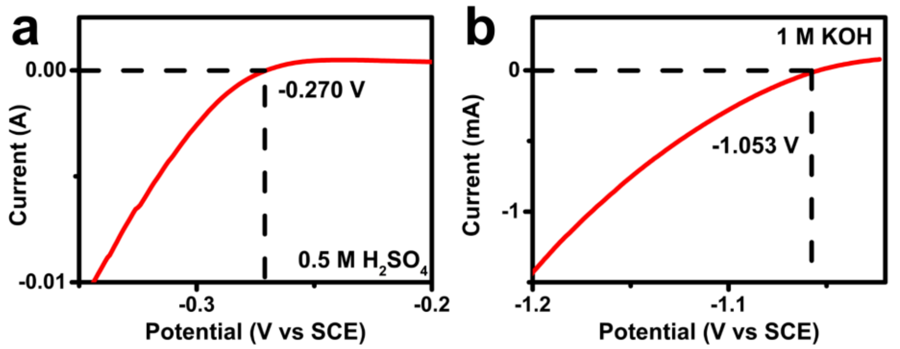

Supplementary Figure 20. Calibration curves for SCE in (a) 0.5 M H_2SO_4 and (b) 1 M KOH.
